# Role of RAGE in obesity-induced adipose tissue inflammation and insulin resistance

**DOI:** 10.1038/s41420-021-00711-w

**Published:** 2021-10-22

**Authors:** Ziqian Feng, Zuoqin Du, Xin Shu, Luochen Zhu, Jiaqi Wu, Qian Gao, Liqun Wang, Ni Chen, Yi Li, Mao Luo, Michael A. Hill, Jianbo Wu

**Affiliations:** 1 Key Laboratory of Medical Electrophysiology of Ministry of Education, Collaborative Innovation Center for Prevention and Treatment of Cardiovascular Disease of Sichuan Province, Drug Discovery Research Center, Southwest Medical University, Luzhou, China; 2 Laboratory for Cardiovascular Pharmacology, Department of Pharmacology, School of Pharmacy, Southwest Medical University, Luzhou, Sichuan China; 3 Zhengzhou Shuqing Medical College, Zhengzhou, Henan China; 4 Dalton Cardiovascular Research Center, University of Missouri, Columbia, Missouri USA

**Keywords:** Fat metabolism, Obesity

## Abstract

Obesity is known to be associated with adipose tissue inflammation and insulin resistance. Importantly, in obesity, the accumulation of proinflammatory macrophages in adipose tissue correlates with insulin resistance. We hypothesized that the receptor for advanced glycation end products (RAGE) and associated ligands are involved in adipose tissue insulin resistance, and that the activation of the AGE–RAGE axis plays an important role in obesity-associated inflammation. C57BL/6J mice (WT) and RAGE deficient (RAGE^−/−^) mice were fed a high fat diet (HFD) and subjected to glucose and insulin tolerance tests. Epdidymal adipose tissue (eAT) was collected and adipose stromal vascular cells isolated using flow cytometry. Visceral adipose tissue macrophage polarization was assessed by quantitative real time PCR. Immunoblotting was performed to evaluate the insulin signaling in adipose tissues. In additional studies, cell trafficking was assessed by injecting labeled blood monocytes into recipient mice. RAGE^−/−^ mice displayed improved insulin sensitivity and glucose tolerance, accompanied by decreased body weight and eAT mass. Exogenous methylglyoxal (MGO) impaired insulin-stimulated AKT signaling in adipose tissues from WT mice fed a normal chow diet, but not in RAGE^−/−^ mice. In contrast, in obese mice, treatment with MGO did not reduce insulin-induced phosphorylation of AKT in WT-HFD mice. Moreover, insulin-induced AKT phosphorylation was found to be impaired in adipose tissue from RAGE^−/−^-HFD mice. RAGE^−/−^ mice displayed improved inflammatory profiles and evidence for increased adipose tissue browning. This observation is consistent with the finding of reduced plasma levels of FFA, glycerol, IL-6, and leptin in RAGE^−/−^ mice compared to WT mice. Collectively the data demonstrate that RAGE-mediated adipose tissue inflammation and insulin-signaling are potentially important mechanisms that contribute to the development of obesity-associated insulin resistance.

## Introduction

Adipose tissue serves as an essential endocrine regulator in glucose homeostasis and is involved in the progression of obesity and type 2 diabetes mellitus (T2DM), at least in part, through the induction of a chronic inflammatory state. Adipose tissue insulin resistance has been associated with dysregulated insulin signaling, impaired glucose uptake, and lipid homeostasis [1, 2]. Advanced glycation end products (AGEs) interact with the receptor for advanced glycation end products (RAGE), (expressed in multiple cell types including adipocytes, macrophages, and endothelial cells) to trigger cell activation and inflammatory responses [3, 4]. RAGE appears to be involved in the progression of obesity, correlating with adipose tissue inflammation, adipocyte hypertrophy, and insulin sensitivity [5, 6]. Supporting a role for ligand activation of RAGE in these metabolic disturbances, a soluble form of RAGE (sRAGE) has been found to competitively bind to AGEs, thus blocking the initiation of the proinflammatory RAGE signaling cascade [7]. Moreover, high-fat feeding elevates the levels of RAGE ligands, HMGB1 and carboxymethyl lysine (CML)-AGE, in the liver and adipose tissue [6].

Studies have suggested that the accumulation of adipose tissue macrophages (ATMs) contributes to local and systemic insulin resistance. The physiology and pathophysiology of ATMs in obesity is, however, a complex process that involves the recruitment of circulating monocytes to white adipose tissue (WAT), a phenotypic switch leading to macrophage polarization [8], and the proliferation of resident macrophages in WAT [9]. It has been reported that RAGE ligands can induce macrophage activation and upregulation of inflammatory factors [10, 11]. In addition, inflammatory responses in adipose tissues are characterized by increased levels of free fatty acids, lipid peroxidation, and oxidative stress, facilitating insulin resistance [12, 13]. Nevertheless, the precise role of RAGE in adipose tissue is currently under appreciated.

Although RAGE mRNA is highly expressed in adipocytes, ATMs, and other adipose tissue cell types the role(s) of RAGE in obesity-associated inflammation and insulin resistance is unclear. Methylglyoxal (MGO) is one of the most potent glycating agents and a major precursor of AGEs [12, 14]. In relation to this, a previous study has demonstrated the MGO-dependent inhibition of insulin receptor-mediated pathways in adipose tissue [15]. Similarly, MGO treatment of 3T3-L1 adipocytes impairs insulin signaling [16]. From a mechanistic standpoint, MGO-derived AGEs may interact with RAGE thereby contributing to the inflammatory microenvironment [17, 18]. In addition, the accumulation of MGO in adipose tissue is associated with increased tissue glycation, further contributing to the development of diabetic complications [16]. Here, we aimed to examine the relative roles of RAGE in the regulation of adipose tissue insulin resistance and ATM phenotype. We found that RAGE deficiency potently reduces the recruitment of circulating monocytes to AT, suppresses M1 macrophage polarization, and improves tolerance to both glucose and insulin. These data demonstrate that the RAGE has the potential to impact physiological and pathophysiological metabolic responses in adipose tissues.

## Results

### RAGE deficiency improved glucose and insulin tolerance

**Fig. 1:**
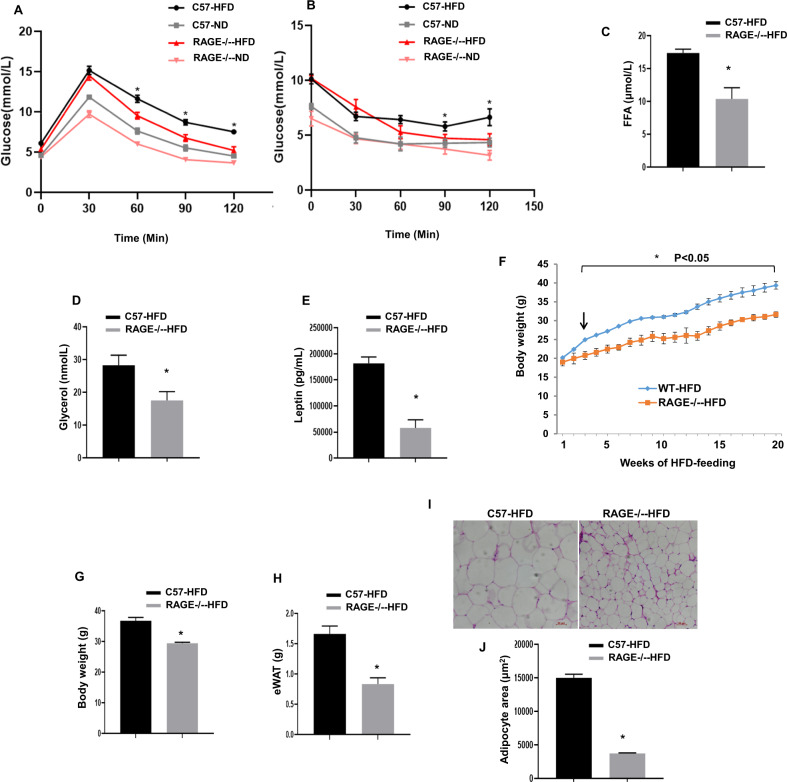
RAGE deficiency improves glucose and insulin tolerance. Mice were fed HFD for 20 weeks. Glucose (GTT) (**A**) and Insulin (ITT) tolerance tests (**B**). *n* = 6 per group. *P < 0.05 vs. WT-ND and RAGE^−/−^-HFDmice. Plasma FFA (**C**), glycerol (**D**), and leptin (**E**) were measured by ELIS. n=6 per group, *P < 0.05 Vs. WT-HFD mice. Temporal changes in body weight (measured weekly) are shown in panel **F** while panel **G** shows the bodyweight after 20 weeks of HFD feeding. *n* = 6 per group; *P < 0.05 Vs. WT-HFD mice. **H** eAT weight was measured (*n* = 6 per group). *P < 0.05 Vs. WT-HFD mice. Data are mean ± SEM. **I** EAT sections were stained by hematoxylin. Representative histological images were obtained from WT-HFD and RAGE^−/−^-HFD mice. Scale bars: 50 μm. **J** The area of adipocyte size is presented as graphs. *n* = 6 per group; *P < 0.05 Vs. WT-HFD mice. All group data are shown as mean ± SEM. FFA, free fatty acid; eAT, epididymal adipose tissue.

To define the role of RAGE in obesity in vivo, we fed mice a high-fat chow diet for 20 weeks, which produced obesity and hyperglycemia. In response to the normal diet, glucose tolerance tests showed ND RAGE^−/−^-ND control mice to have a moderate improvement in glucose metabolism when compared to the WT-ND mice. (Fig. 1A) Obese, RAGE^−/−^-HFD mice showed significantly improved glucose tolerance compared with WT mice fed the HFD (Fig. 1A). Following exogenous insulin injection, glucose levels were similar in RAGE^−/−^-ND and WT-ND mice. In contrast, compared with WT-HFD, exogenous insulin caused a significant reduction in blood glucose levels in RAGE^−/−^-HFD mice at both 90 and120 min (Fig. 1B). Based on these changes, we further examined the relative metabolic phenotypes of the HFD-fed groups. RAGE^−/−^ mice showed lower concentrations of plasma free fatty acids (FFA) and glycerol (Fig. 1C, D) and decreased circulating levels of leptin (Fig. 1E) compared to WT mice similarly treated with 20 weeks of HFD. These metabolic results are thus consistent with RAGE deficiency being associated with improved glucose tolerance and insulin sensitivity in HFD-induced obesity in mice.

We further determined differences in body and visceral AT weights after 20 weeks of HFD feeding. WT-HFD mice displayed a significantly increased body weight compared to RAGE^−/−^-HFD mice (Fig. 1F, G). Bilateral eAT weights were significantly lower in RAGE^−/−^-HFD mice than that in WT-HFD mice (Fig. 1H). Further, epididymal adipocyte area was decreased significantly in RAGE^−/−^-HFD mice compared with that of WT-HFD mice (Fig. 1I, J). Thus, RAGE appeared to play an important role in the development of obesity and was further related to glucose homeostasis.

### RAGE deficiency decreases the accumulation of adipose tissue macrophages

**Fig. 2:**
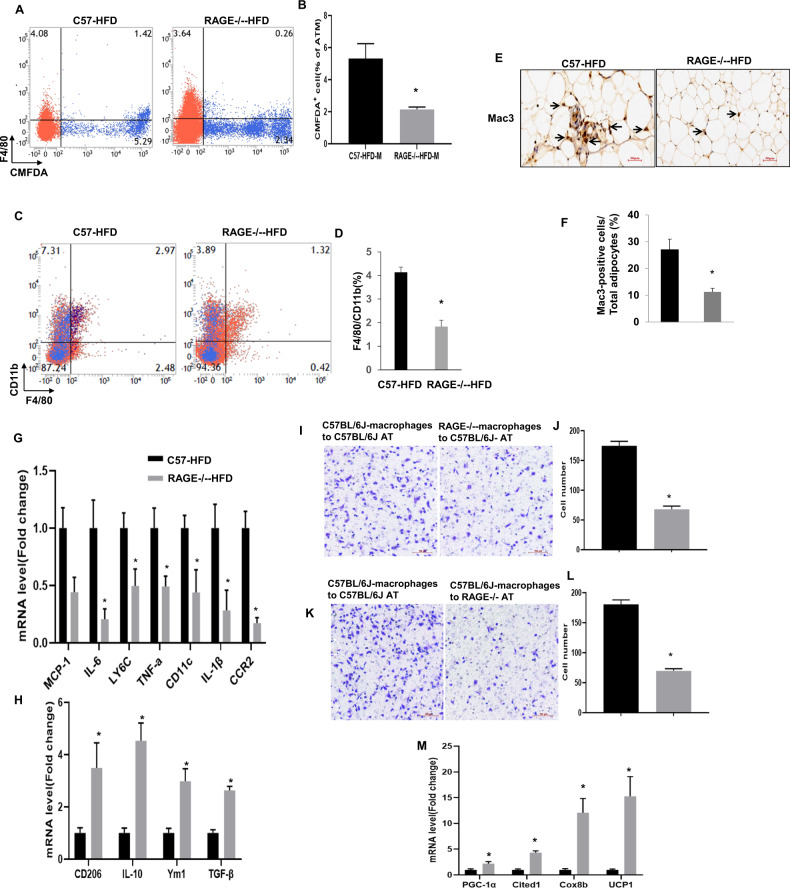
RAGE deficiency decreases the accumulation of adipose tissue macrophages. Quantitation by flow cytometry of the proportion of CMFDA+ ATMs (**A**, **B**) and CD11b^+^F4/80^+^ (**C**, **D**) in the SVF of eAT from WT-HFD and RAGE^−/−^-HFD mice. *n* = *6* per group; *P < 0.05 Vs. WT-HFD mice. **E**, **F** Immunohistochemical detection of Mac3 in eAT of from HFD mice. *n* = 6 per group; *p < 0.05 Vs. WT-HFD mice. Magnification, ×200, is indicated as the ratio of Mac3-positive cells to total cells. Scale bars: 50 μm. **G**, **H** Quantitative RT-PCR analysis of total RNA isolated from eAT for IL-6, IL-1β, TNF-α, MCP-1, and CD11c, IL-10, YM1, TNF-β, and CD206 mRNAs. Data were normalized by the amount of 18s mRNA and expressed relative to the corresponding WT-HFD. *n* = 6 per group. *P < *0.05* Vs. WT-HFD mice. **I**, **J** Isolated peritoneal macrophages as indicated were added to upper chambers. eAT from either WT-HFD or RAGE^−/−^-HFD mice were chopped into approximately 1 mm pieces, and then 50 mg of chopped eAT were placed in the lower chambers. After 6 h of incubation, cells were fixed and stained with crystal violet. Migrated cells were counted. Representative images of cell migration are shown. **K**, **L** Quantitative assessment of triplicate migration experiments was performed. *P <0.05 Vs. WT-HFD mic-derived eAT. **M** *Ucp1*, *Pgc1a*, *Cited1*, and *Cox8b* mRNA expression levels in the eAT of WT-HFD and RAGE^−/−^-HFD mice. *n* = 6 per group; *P < *0.05* Vs. WT-HFD mice. All group data are shown as mean ± SEM.

Since genetic deficiency of RAGE has been previously shown to prevent the effects of HFD on adipose tissue inflammation [6], we next investigated whether the accumulation of ATMs was involved in the recruitment of circulating monocytes to WAT together with a phenotypic switch in macrophages polarization. We directly measured circulating monocyte migration into eAT using an in vivo macrophage tracking technique. With this approach, isolated blood monocytes were obtained from normal diet C57BL/6J donor mice and labeled with CMFDA-Green dye. The labeled monocytes were then injected into recipient RAGE^−/−^-HFD and WT-HFD mice. FACS analysis was then performed on the stromal vascular fraction (SVF) of eAT. The results showed that the total number of CMFDA+ ATMs was markedly lower in eAT from RAGE deficient HFD mice compared to WT-HFD mice (Fig. 2A, B). In addition, the fraction of ATMs (CD11b^+^F4/80^+^) was decreased in eAT from RAGE deficient mice under HFD conditions (Fig. 2C, D). We next examined the number of infiltrated macrophages in adipose tissue by immunohistochemical analysis using the Mac3 antibody. The degree of macrophage infiltration was assessed by calculating the ratio of infiltrated macrophages to total cells in eAT. The result showed that Mac3-positive macrophage infiltration was decreased significantly in RAGE^−/−^-HFD mice compared with WT-HFD mice (Fig. 2E, F).

We further evaluated ATM polarization in eAT by qRT-PCR. As shown in Fig. 2G, the mRNA levels of pro-inflammatory genes, including IL-6, IL-1β, TNF-α, MCP-1, and CD11c, were significantly decreased in eAT from RAGE^−/−^ -HFD mice compared with WT-HFD mice, indicating that RAGE deficiency significantly down-regulates M1 macrophage pro-inflammatory genes. Similarly, compared with WT-HFD mice, RAGE^−/−^-HFD mice exhibited up-regulation the mRNA levels M2 markers IL-10, YM1, TNF-β, and CD206 (Fig. 2H).

To extend observations relating to chemotaxis, an alternate macrophage migration assay was performed using a transwell culture system. Peritoneal macrophages were isolated from either WT-HFD or RAGE^−/−^-HFD mice. Representative macrophage images shown in Fig. 2I–L, Quantitative analysis revealed that the migration of RAGE^−/−^-HFD-derived macrophages towards eAT explants obtained from WT-HFD was significantly impaired compared with that of WT-HFD-derived macrophages. Similarly, under macrophage migration towards eAT obtained from RAGE^−/−^-HFD mice, RAGE^−/−^-HFD-derived macrophages exhibited a significant reduction in migration compared with macrophage from WT-HFD mice. As the browning of white adipose tissues increases the metabolic rate and improves insulin resistance [19, 20] markers of browning were determined in eAT from WT and RAGE^−/−^-HFD mice. Thus, as shown, RAGE^−/−^-HFD mice exhibited significantly higher levels of the Ucp1, Pgc1a, Cited1, and Cox8b mRNAs than WT-HFD mice in eAT (Fig. 2M). Collectively, these findings support our observation that RAGE deficiency directly impairs ATMs chemotaxis and recruitment.

### RAGE Deficiency Protects MGO-inhibited Insulin Signaling in Adipose Tissue

**Fig. 3:**
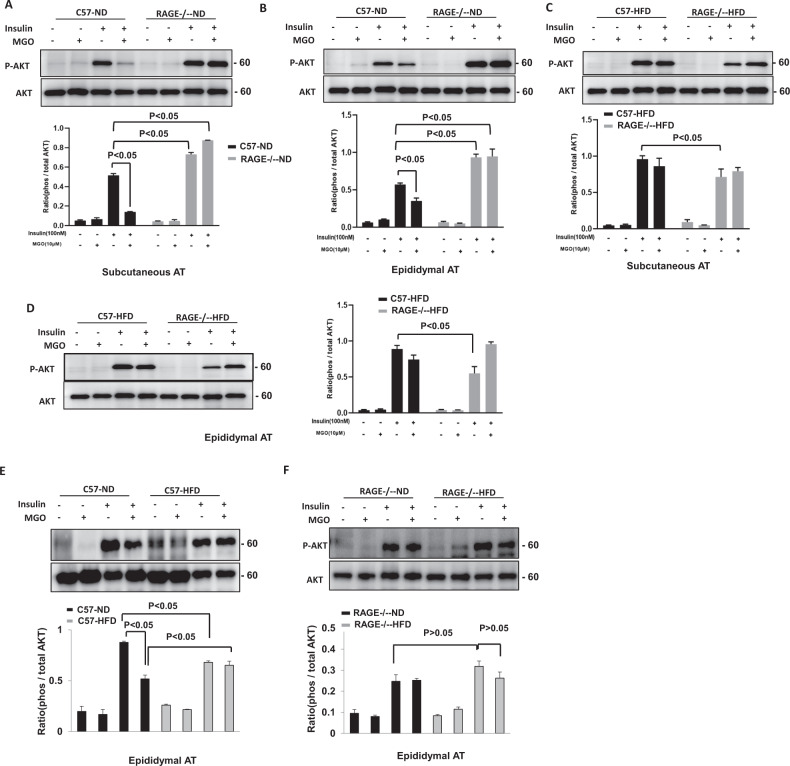
RAGE deficiency protects MGO-inhibited insulin signaling in adipose tissue. The treatment of insulin (100nM) for 10 mins stimulated Ser 473 phosphorylation of AKT after pretreatment with MGO (10 µM) for 16 h in AT explants from WT and RAGE^−/−^ mice. Representative immunoblots and quantification of subcutaneous AT (sAT) (**A**) and eAT (**B**) from normal diet (ND) mice. Representative immunoblots and quantification of sAT (**C**) and eAT (**D**) from HFD mice as indicated. **E**, **F** Comparison of AKT phosphorylation from either ND or HFD mice with the same strain as indicated. All graphs correspond to the adjacent blots above and represent densitometric analyses of 3 independent experiments. *P*-values indicating the significance of difference are indicated in the respective bar diagrams. All group data are shown as mean ± SEM.

It has been shown previously that MGO impairs insulin sensitivity through reducing insulin-induced AKT phosphorylation in endothelial cells [21]. We therefore examined whether RAGE plays a role in mediating insulin sensitivity in subcutaneous AT (sAT) and epididymal adipose explants. Insulin treatment (100 nM; 10 mins) stimulated Ser 473 phosphorylation of AKT in AT explants from WT and RAGE^−/−^ mice. The levels of AKT phosphorylation were found to be significantly higher in sAT (Fig. 3A) and eAT (Fig. 3B) in RAGE^−/−^-ND mice than in WT-ND mice. In contrast, AKT phosphorylation levels in sAT (Fig. 3C) and eAT (Fig. 3D) from high fat diet-fed WT and RAGE^−/−^ mice were similar. After 20 weeks of the high-fat diet, the phosphorylation levels of AKT in ATs of RAGE^−/−^-HFD mice were significantly diminished in response to insulin stimulation compared with those noted in WT-HFD mice. Furthermore, as shown in Fig. 3, we found that pretreatment of MGO (10 µM) for 16 h markedly inhibited insulin-stimulated the phosphorylation of AKT in AT in WT mice, but not in RAGE^−/−^ mice. Interestingly, in obesity, the treatment of MGO is associated with defective inhibition of insulin-stimulated AKT in adipose tissue.

We further examined the phosphorylation of AKT in eAT from WT and RAGE^−/−^ mice, fed either ND or HFD. As shown in Fig. 3E, compared with C57BL/6J-ND, insulin significantly diminished the phosphorylation of AKT in eAT from C57BL/6J- HFD mice, which is consistent with the previous study [22]. Pretreatment with MGO inhibited insulin-stimulated AKT phosphorylation in C57BL/6J-ND mice but not in C57BL/6J-HFD mice. Obesity-induced insulin resistance results in a reduction in insulin-AKT phosphorylation while insulin-stimulated phosphorylation of AKT in eAT was slightly increased from RAGE^−/−^-HFD mice compared with RAGE^−/−^-ND mice (*P* > 0.05; Fig. 3F). MGO exhibited a partial inhibitory effect on insulin-stimulated AKT phosphorylation in RAGE^−/−^-HDF mice (*P* > 0.05; Fig. 3F), but not in RAGE^−/−^-ND mice. Taken together, these results suggest that RAGE plays a key role in the regulation of the metabolic effects of insulin in adipose tissues.

## Discussion

**Fig. 4:**
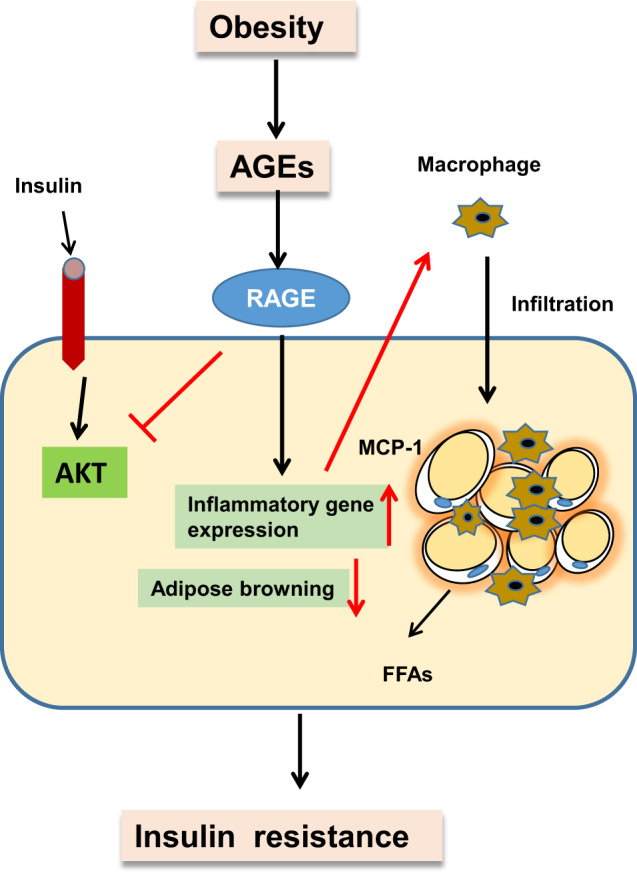
Proposed model by which RAGE mediates glucose homeostasis in adipose tissue. Schematic diagram illustrating potential mechanisms by which RAGE affects systemic glucose homeostasis through inhibition of circulating monocyte recruitment to visceral AT and downregulation of the M1 population of macrophages. In the absence of RAGE signaling, decreased FFA production and enhanced AT browning are also suggested to provide a protective effect against increased systemic insulin resistance. FFAs, free fatty acids.

The present study demonstrates the involvement of RAGE in obesity-mediated adipose tissue inflammation and insulin resistance. Consistent with this, RAGE^−/−^ mice exhibited reduced fat mass and improved insulin resistance in HFD-induced obesity. Further, RAGE deficiency decreased the accumulation of macrophages in adipose tissue. On the basis of our data and published studies, we propose that RAGE and its ligands, such as MGO, contribute to the regulation of eAT macrophages and insulin resistance as schematically illustrated in Fig. 4. Supporting this scheme, 1. FACS analysis showed reduced recruitment of circulating monocytes to visceral AT in RAGE^−/−^ mice; 2. RAGE deficiency attenuated MGO- mediated inhibition of insulin signaling in adipose tissue; and 3. gene expression profiling studies in adipose tissues in RAGE^−/−^ mice revealed an up-regulation of M1-type polarization and white AT browning, potentially linked to the decreased plasma FFA and leptin levels. Collectively, these findings highlight an important role for RAGE in obesity-related insulin resistance.

Earlier studies have suggested that RAGE is required for the development of diet-induced obesity and its associated pathologies of insulin resistance [6, 23]. In the present studies, we used mice genetically deficient in RAGE to examine the role of RAGE in macrophage recruitment and metabolic function. FACS analysis of adipose tissue showed that RAGE^−/−^ mice on high-fat diets have reduced macrophage numbers compared with WT mice. RT-PCR further showed decreased adipose tissue gene expression of M1 polarization markers in RAGE^−/−^ mice. Our results contrast with the recent report of reduced adipose tissue macrophages in obese RAGE^−/−^ mice [6]. Using peritoneal mouse macrophages, we demonstrated that RAGE^−/−^ mice-derived AT was associated with decreased WT macrophage migration. Meanwhile, we showed that the migration of RAGE^−/−^ mice-derived macrophages, towards AT, was significantly reduced compared with WT mice-derived macrophages. Collectively these observations provide evidence that RAGE is critical for macrophage recruitment and polarization. Further studies are required to clarify how macrophage-mediated chronic inflammation in WAT contributes to insulin resistance in RAGE^−/−^ mice. In addition, white AT browning is associated with increased energy expenditure and insulin resistance improvement [24]. Previous studies have reported that RAGE deficient mice fed HFD exhibit increased metabolic rate [6], and white adipose tissue of the recipients of the RAGE-deleted adipocytes displayed increased expression of UCP1 protein through paracrine mechanisms [25]. Thus, our data are consistent with a mechanism whereby RAGE-regulated improvements in insulin resistance likely involve adipose tissue browning and direct effects on AT to inhibit inflammation.

Our studies further provided evidence that the RAGE ligand, MGO, impairs insulin-mediated signaling action via RAGE in adipose tissue explants from mice on a standard chow diet. Phosphorylation levels of AKT in adipose tissues from RAGE^−/−^-HFD mice were significantly diminished in response to insulin stimulation compared with those in WT mice. In contrast, AKT phosphorylation levels were significantly increased in adipose tissues of normal-diet RAGE^−/−^ mice. According to the results of the current study, decreased bodyweight together with improved glucose and insulin tolerance in RAGE^−/−^ mice, the absence of RAGE may conceivably reduce the risk of developing diabetes in mice. However, the decrease in insulin-stimulated AKT activation in AT from RAGE^−/−^*-*HFD mice does not correlate with the results of whole-body glucose homeostasis. Improved insulin sensitivity from RAGE^−/−^*-*HFD mice is unlikely to be explained by lower insulin-stimulated AKT in AT, which demonstrated that high-fat diet-induced insulin resistance in RAGE deficient adipose tissue does not coincide with a reduction in AKT phosphorylation. Monden et al. reported that overexpression of RAGE attenuated insulin signaling associated with suppression of glucose transporter type 4 in cultured adipocytes [5]. Future studies with adipose-specific RAGE-deficient mice are required to improve our understanding of how ATMs contribute to whole-body insulin resistance and glucose handling.

The differences in insulin sensitivity between the RAGE^−/−^ and WT mice were augmented on the feeding of the high-fat diet. Obesity-induced insulin resistance results in a reduction in insulin-AKT phosphorylation. While the phosphorylation levels of AKT in adipose tissues of RAGE^−/−^ mice were significantly diminished in response to insulin stimulation compared with those noted in WT mice. Thus, it is postulated that RAGE–deficient mice appear to show tissue-specific insulin sensitivity on a high-fat diet. The effect observed in adipose tissue could result from some compensatory mechanism such as some other insulin signaling or could be due to the overall improved insulin sensitivity of the RAGE–deficient mice. It was assumed that this effect was noted due to these mice’s other insulin-sensitive tissues, liver, and skeletal muscle. Indeed, RAGE neutralizing antibody significantly prevented muscle atrophy and dysfunction in diabetic mice via activating the phosphorylation of AKT [26]. The underlying mechanism requires further investigation. Further animal and cellular experiments are required to substantiate the effects of RAGE on glucose metabolism and delineate the mechanisms underlying the association of T2DM.

As mentioned earlier, it is notable that other studies have shown contrasting results regarding TNF-α mRNA levels in AT between RAGE deficient and WT mice fed HFD [5, 6]. While the exact reasons for these differences are uncertain there were methodological differences in HFD composition and time between the present work (60% of HFD for 20 weeks) and previous studies. The extent to which diet and feeding regime might have accounted for such differences in ATMs phenotypes remains to be established. A further caveat is that we present data only for male mice. Future studies should include studies of females to account for potential sexual dimorphism.

## Conclusion

In the current study, we provide evidence that RAGE deficiency suppresses the number of ATMs and favors the anti-inflammatory M2 phenotype (as opposed to inflammatory M1 polarization) in an HFD-induced model of obesity (Fig. 4). RAGE deficient mice further showed improved glucose tolerance and insulin sensitivity in obese mice and which was associated with insulin-AKT signaling. Collectively the data support an important role for RAGE in inflammation and insulin resistance in adipose tissue.

## Materials And Methods

### Animals

6-8 weeks old C57BL/6J mice were obtained from the Chongqing Medical University Animal Center (Chongqing, China). RAGE^−/−^ mice were purchased from the Jackson Laboratory (Bar Harbor, ME, USA). All protocols for animal use were reviewed and approved by the Animal Care Committee of Southwest Medical University in accordance with Institutional Animal Care and Use Committee guidelines.

### HFD-fed mouse model

Eight-week-old male C57BL/6J mice and RAGE^−/−^mice were fed a high-fat diet (HFD) (TP2330055A; in calories, fat 60%, carbohydrate 25%, and protein 15%; Research Diet, Trophic Animal Feed High-tech Co. Ltd, China) for 20 weeks, as described previously [19]. Age-matched male mice that were fed a standard chow diet (ND; TP2330055AC; in calories fat 10%, carbohydrate 75%, and protein 15%; Research Diet, Trophic Animal Feed High-tech Co. Ltd, China) were used as controls. Blood samples were obtained from the tail vein and blood glucose levels were measured using an automated glucometer (Accu-Chek; Roche Diagnostics, Mannheim, Germany). Bodyweight was monitored every seven days. At the end of the studies, blood was collected from the inferior vena cava and centrifuged at 1500 *× g* for 10 min for measurement of plasma FFAs, glycerol, and leptin.

### Glucose and insulin tolerance tests

Following a 4hr fast glucose (GTT) and insulin (ITT) tolerance tests were performed in response to intraperitoneal (IP) injection of D-glucose (Roth, Karlsruhe, Germany) (2 g of glucose/kg body mass) or insulin (0.75 U insulin/kg body mass, respectively. Blood samples were obtained from the tail vein, and whole blood glucose levels were measured at 0, 30, 60, and 120 min using One Touch® Vita® glucometer (Lifescan, Zug, Switzerland).

### Plasma parameters

Plasma leptin concentrations were measured using a commercially available ELISA kit (ThermoFisher Scientific, Massachusetts, USA). Plasma FFA and glycerol levels were measured by specific assay kits (Abcam, USA).

### Monocyte isolation

Blood monocytes were isolated from ND-fed-C57BL/6J mice using an EasySep Mouse Monocyte Enrichment kit (Stem Cell Technologies, Massachusetts, USA) as previously described^20^. Monocyte subsets were enriched with the EasySep kit according to the manufacturer’s instructions.

### In vivo trafficking

The isolated blood monocytes were stained with CellTracker™ Green CMFDA (ThermoFisher Scientific) as previously described [14], and ~8 x 10^6^ cells were suspended in 0.2 mL PBS to be injected into the tail vein of each group of mice. 12 h after the injection, the ATMs were immediately isolated from epdidymal adipose tissue (eAT) and analyzed via flow cytometry.

### Flow cytometric analysis (FACS)

FACS was performed using a FACS Canto II (BD Biosciences) as previously described [19]. Adipose stromal vascular cells were prepared from collagenase digested adipose tissue. For macrophage characterization antibodies to cell surface antigens included F4/80, Ly6C, CD11b, and CD11c (eBioscience, San Diego, California). Numbers obtained were represented as the percentage of the highest subsets. Gating strategy is shown in Supplementary Fig. 1.

### Isolation of peritoneal macrophages

ND-fed C57BL/6J mice were intraperitoneally injected with 1 mL of a 3% sterile thioglycollate solution (Difco Laboratories, Maryland, USA). After 4 days, peritoneal cells were harvested by washing the peritoneal cavity with sterile PBS. Macrophages from the peritoneum were then counted by microscopy.

### Migration

Coculture experiments were conducted as described previously [21]. In brief, to assess peritoneal macrophage migration, eAT was minced into approximately 1 mm pieces with 50 mg of eAT explants then being placed in the lower chambers of Transwell plates (0.4 μm pore size) and cocultured with an equal volume of peritoneal macrophages in the upper chambers. Cells that migrated to the lower chamber were counted.

### Histological analysis

Mouse eAT was isolated, fixed, and embedded in paraffin, and serially sectioned (6 µm). Cross-sections were prepared for immunohistochemistry by incubating overnight at 4 °C with a 1:100 dilution of an antibody to mouse Mac3 (M3/84; BD Biosciences). Immune complexes were detected using biotinylated secondary antibodies (BD Biosciences), HRP-conjugated streptavidin (Dako, California, USA), and the peroxidase substrate diaminobenzidine (Dako). Images were captured using a microscope (Leica, Wetzlar, Germany). Macrophages in eAT were quantified by calculating the ratio of nuclei of Mac3-positive cells to total nuclei in 10 fields of 3 slides for each individual mouse using 6 mice for each group [27]. For quantifying adipocyte area, 5–6 fields per section were averaged with 6 mice per group being studied. Image J software (NIH, Maryland, USA) was used to measure adipocyte area, and the data are shown as the average cell area (in μm^2^).

### Quantitative real-time PCR

eAT was collected and total RNA extracted using TRIzol reagent (Invitrogen, California, USA). qRT-PCR was performed using miScript SYBR Green PCR Kits (Qiagen). Each sample was analysed in duplicate with ribosomal 18S RNA as an internal control. Fold changes in gene expression were determined using the 2^−ΔΔCT^ method and values are presented as the mean ± SEM. All primers are listed in Supplemental Table 1.

### Immunoblotting

Adipose tissues lysates were prepared, and equal amounts of protein were subjected to SDS-polyacrylamide electrophoresis and transferred to polyvinylidene difluoride membranes by electroblotting. After blocking, the membranes were incubated with antibodies directed against phospho-Akt (Ser473), total AKT (Cell Signaling Technology, Massachusetts, USA). The secondary antibody was horseradish-peroxidase (HRP)-conjugated goat IgG raised against IgG (Santa Cruz Biotechnology). Blots were developed with ECL substrate (Pierce).

### Statistical analysis

Data are presented as the mean ± SEM of triplicate experiments. Two-tailed Student’s *t*-tests were used when comparing two experimental groups and analysis of variance (ANOVA) for multiple comparisons.

## Supplementary information

Supplemental Figure legends

Supplemental Figure 1

Supplemental table 1

## Data Availability

All data generated or analyzed during this study are included in this article.
