# Supplementary material for: Role of RAGE in obesity-induced adipose tissue inflammation and insulin resistance
Source: Cell Death Discov. 2021 Oct 22;7:305. doi: 10.1038/s41420-021-00711-w (PMC8536716; doi:10.1038/s41420-021-00711-w)
Supplement: Supplementary file 1 — Supplemental Figure legends [file 41420_2021_711_MOESM1_ESM.docx]

**Supplementary Figure legends**

**Supplementary Figure 1.** Gating Strategy for Figure 2 in the SVF of eAT from WT-HFD (A) and RAGE^-/-^-HFD mice (B).
