# Supplementary figures and images for: Role of RAGE in obesity-induced adipose tissue inflammation and insulin resistance
Source: Cell Death Discov. 2021 Oct 22;7:305. doi: 10.1038/s41420-021-00711-w (PMC8536716; doi:10.1038/s41420-021-00711-w)

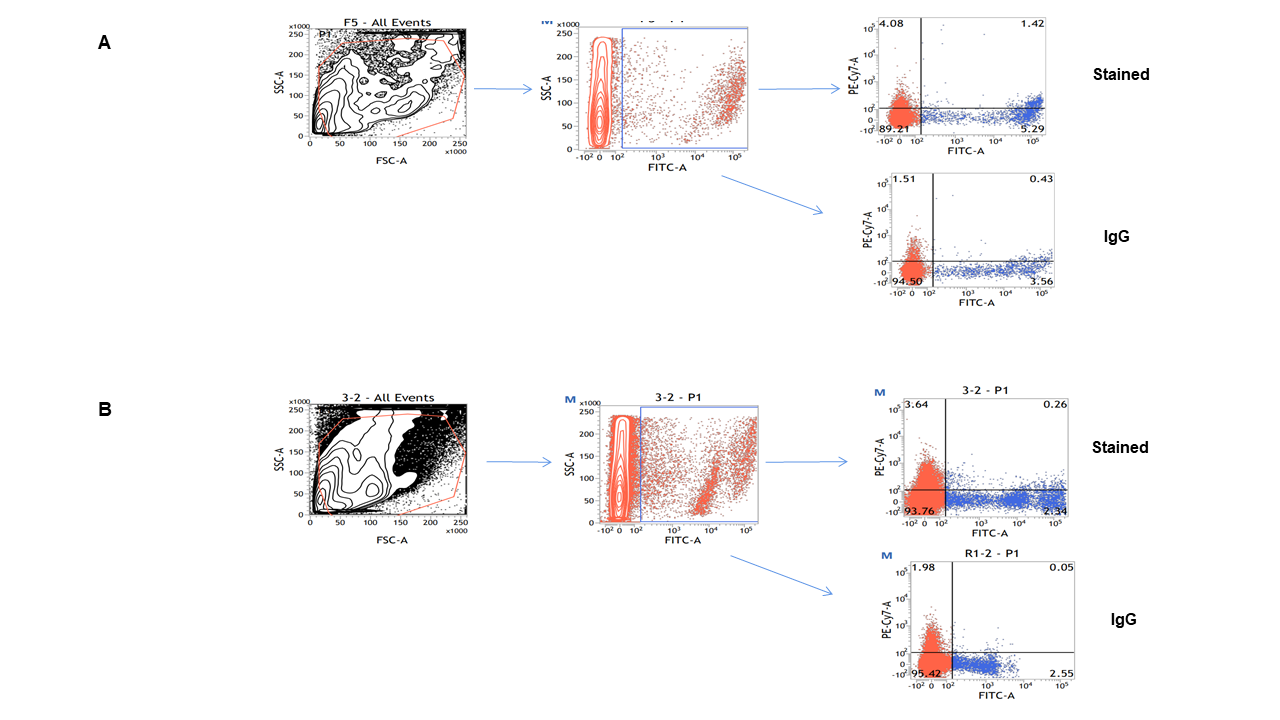

Supplement: Supplementary file 2 — Supplemental Figure 1 [file 41420_2021_711_MOESM2_ESM.tif]
