# Supplementary material for: Role of RAGE in obesity-induced adipose tissue inflammation and insulin resistance
Source: Cell Death Discov. 2021 Oct 22;7:305. doi: 10.1038/s41420-021-00711-w (PMC8536716; doi:10.1038/s41420-021-00711-w)
Supplement: Supplementary file 3 — Supplemental table 1 [file 41420_2021_711_MOESM3_ESM.docx]

# Table 1 Sequences of primers used in the study

| Gene  (Accession number) |  | Primer |
| --- | --- | --- |
| CCR2  (NM_009915) | Forward  Reverse | GCAAGTTCAGCTGCCTGCAA  ATGCCGTGGATGAACTGAGGTAA |
| Ly6C  (NM_001252058) | Forward  Reverse | ACTGTGCCTGCAACCTTGTC  CACACAGTAGGGCCACAAGA |
| IL-6  (NM_031168) | Forward  Reverse | CACATGTTCTCTGGGAAATCG  TTGTATCTCTGGAAGTTTCAGATTGTT |
| TNF-α  (NM_001124357) | Forward  Reverse | ACGGCATGGATCTCAAAGAC  AGATAGCAAATCGGCTGACG |
| CD11c | Forward | CACTCAGTGACTGCCCAAAA |
| ([NM_021334.2](https://www.ncbi.nlm.nih.gov/entrez/viewer.fcgi?db=nucleotide&id=118130485)) | Reverse | CCTCAAGACAGGACATCGCT |
| IL-1β | Forward | ACTACAGGCTCCGAGATGAACAAC |
| ([NM_008361.4](https://www.ncbi.nlm.nih.gov/entrez/viewer.fcgi?db=nucleotide&id=921274059)) | Reverse | CCCAAGGCCACAGGTATTTT |
| MCP1  (NM_011333.3) | Forward  Reverse | ACGCAGGTCCCTGTCATG  GTTCACTGTCACACTGGTCA |
| Ym1   NM_009892.3 | Forward  Reverse | AGAGTGCTGATCTCAATGTGG  GGGCACCAATTCCAGTCTTAG |
| IL-10 | Forward | TGTCAAATTCATTCATGGCCT |
| ([NM_010548.2](https://www.ncbi.nlm.nih.gov/entrez/viewer.fcgi?db=nucleotide&id=291575143)) | Reverse | ATCGATTTCTCCCCTGTGAA |
| TGFβ1 | Forward | TGCTAATGGTGGACCGCAA |
| ([NM_011577.2](https://www.ncbi.nlm.nih.gov/entrez/viewer.fcgi?db=nucleotide&id=930697458)) | Reverse | CACTGCTTCCCGAATGTCTGA |
| CD206 | Forward | CATGGATGTTGATGGCTACTGGAG |
| ([NM_008625.2](https://www.ncbi.nlm.nih.gov/entrez/viewer.fcgi?db=nucleotide&id=224967061)) | Reverse | GTCTGTTCTGACTCTGGACACTTG |
| 18S rRNA | Forward | GCAATTATTCCCCATGAACG |
| (NR_003278.3) | Reverse | GGCCTCACTAAACCATCCAA |
